# Pharmacokinetic study of capivasertib and the CYP3A4 substrate midazolam in patients with advanced solid tumors

**DOI:** 10.1007/s00280-024-04667-3

**Published:** 2024-04-20

**Authors:** Claire Miller, Roberto Sommavilla, Cindy L. O’Bryant, Minal Barve, Afshin Dowlati, Jason J. Luke, Mahmuda Khatun, Thomas Morris, Marie Cullberg

**Affiliations:** 1 Clinical Pharmacology and Quantitative Pharmacology, BioPharmaceuticals R&D, AstraZeneca, Cambridge, UK; 2 Late Developmental Oncology, AstraZeneca, Cambridge, UK; 3 University of Colorado Cancer Center, Aurora, CO USA; 4 Mary Crowley Cancer Research Centers, Dallas, TX USA; 5 University Hospitals Seidman Cancer Center and Case Western Reserve University, Cleveland, OH USA; 6 UPMC Hillman Cancer Center, Pittsburgh, PA USA; 7 Clinical Pharmacology and Quantitative Pharmacology, BioPharmaceuticals R&D, AstraZeneca, Gothenburg, Sweden

**Keywords:** Capivasertib, AKT inhibitor, Midazolam, CYP3A4

## Abstract

**Purpose:**

Capivasertib, a potent, selective inhibitor of all three AKT serine/threonine kinase (AKT) isoforms, is being evaluated in phase 3 trials in advanced breast and prostate cancer. This study evaluated the drug–drug interaction risk of capivasertib with the cytochrome P450 3A substrate midazolam in previously treated adults with advanced solid tumors.

**Methods:**

Patients received oral capivasertib 400 mg twice daily (BID) on an intermittent schedule (4 days on/3 days off) starting on day 2 of cycle 1 (29 days) and on day 1 of each 28-day cycle thereafter. In cycle 1 only, patients received oral midazolam (1 mg) on day 1 (alone), and days 8 and 12 (3rd day off and 4th day on capivasertib, respectively). Midazolam pharmacokinetics on days 8 and 12 were analyzed versus day 1. Capivasertib, with or without standard-of-care treatment, was continued in patients deemed likely to benefit. Safety and exploratory efficacy analyses were conducted.

**Results:**

Capivasertib–midazolam coadministration increased midazolam exposure (*n* = 21): geometric mean ratio (90% confidence interval) AUC_inf_ and *C*_max_ was 1.13 (0.97–1.32) and 1.15 (0.99–1.33) for day 8 versus day 1, and 1.75 (1.50–2.05) and 1.25 (1.08–1.46) for day 12 versus day 1. The capivasertib safety profile was manageable when administered with or without midazolam. Two patients had partial responses to treatment.

**Conclusion:**

The up to 1.75-fold increase in midazolam exposure indicates capivasertib is a weak CYP3A inhibitor at 400 mg BID on an intermittent schedule. Capivasertib was well tolerated; exploratory efficacy analysis demonstrated evidence of clinical activity in this heavily pre-treated population.

ClinicalTrials.gov: NCT04958226.

**Supplementary Information:**

The online version contains supplementary material available at 10.1007/s00280-024-04667-3.

## Introduction

The phosphoinositide 3-kinase (PI3K)/AKT serine/threonine kinase (AKT) pathway regulates cell proliferation, growth, and survival, and is frequently dysregulated in cancer [1, 2]. AKT, expressed in three isoforms (AKT1–3), is a central node in this signaling pathway [2, 3].

Capivasertib is a potent, selective inhibitor of all three isoforms of AKT [4]. Results from an early dose-escalation trial informed the recommended dosing schedule that is being used in the majority of ongoing phase 3 trials of capivasertib combination therapy: capivasertib administered orally twice daily (BID) in an intermittent dosing schedule (4 days on, 3 days off) [5].

The CAPItello-291 double-blind, phase 3 randomized trial demonstrated that adding capivasertib 400 mg BID given 4 days on, 3 days off to fulvestrant significantly improved progression-free survival (PFS) in patients with hormone receptor-positive, human epidermal growth factor receptor 2-negative advanced breast cancer that had progressed on prior aromatase inhibitor therapy with or without a cyclin-dependent kinase 4/6 inhibitor; the addition of capivasertib to fulvestrant significantly improved the dual primary endpoints of PFS in the overall population (hazard ratio 0.60; 95% confidence interval [CI] 0.51–0.71; *p* < 0.001), and in patients with *PIK3CA/AKT1/*phosphatase and tensin homolog (*PTEN*)-altered tumors (hazard ratio 0.50; 95% CI 0.38–0.65; *p* < 0.001) compared with placebo plus fulvestrant. Exploratory analyses in the *phosphatidylinositol-4,5-bisphosphate 3-kinase catalytic subunit alpha* (*PIK3CA*) */AKT1/PTEN*-nonaltered population show capivasertib has activity beyond *PIK3CA/AKT1/PTEN*-altered tumors. Capivasertib plus fulvestrant was generally well tolerated; the discontinuation rate due to adverse events (AEs) was low, and the most common AEs were diarrhea, rash, and nausea [6]. Data from CAPItello-291 led to the first regulatory approval of capivasertib plus fulvestrant in patients with tumors harboring one or more biomarker alterations (*PIK3CA*, *AKT1*, or *PTEN*) [7]. The efficacy and safety profile of capivasertib has also been demonstrated in patients with advanced solid tumors in other settings, including in combination with other antitumor agents [8–10].

Many oral, small-molecule drugs are substrates for cytochrome P450 3A (CYP3A) metabolic enzymes, including anticancer agents used to treat patients with advanced cancer and other drugs that may be used to manage comorbid conditions [11]. Results from in vitro studies have shown that capivasertib has the potential to act as a reversible and time-dependent inhibitor of the CYP3A metabolic pathway (unpublished data). As capivasertib is likely to be administered concomitantly with CYP3A substrates in clinical practice, it is essential to determine any potential interaction risk. This phase 1 study was designed to investigate the interaction potential by evaluating the effect of repeated doses of capivasertib (at the therapeutic dose and schedule pursued in clinical development) on the pharmacokinetics (PK) of the sensitive CYP3A substrate midazolam in patients with advanced solid tumors.

## Materials and methods

### Study design and objectives

**Fig.1:**
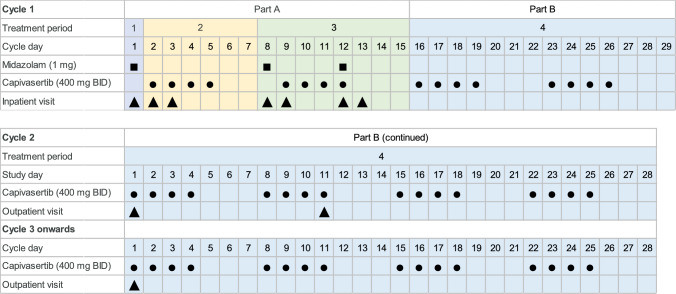
Study design. In part A, all patients received capivasertib BID (4 days on/3 days off); at the investigator’s discretion, patients with estrogen receptor-positive breast cancer could also receive intramuscular fulvestrant 500 mg on day 1 and day 15 of cycle 1, and day 1 of each cycle thereafter. In part B of the study, at the investigator’s discretion, patients were recommended to receive capivasertib in combination with standard-of-care treatment. BID, twice daily

This phase 1, multicenter (5 sites in the USA), open-label, fixed-sequence study (NCT04958226) involved patients with advanced solid tumors. The study comprised two parts (parts A and B). Part A was conducted to evaluate the effect of capivasertib monotherapy on the PK of midazolam, and consisted of a screening period of up to 28 days and three treatment periods, all occurring in the first half of cycle 1 (29 days). Treatment period 1 was midazolam alone (1 mg) administered on day 1 of cycle 1, treatment period 2 was days 2–7 of cycle 1 with capivasertib 400 mg BID dosing on an intermittent schedule (4 days on/3 days off), and treatment period 3 was days 8–15 of cycle 1 with midazolam (1 mg) administered on day 8 and day 12 (corresponding to the 3rd day off and 4th day on capivasertib, respectively; Fig. 1). In part A, at the investigator's discretion, patients with estrogen receptor-positive breast cancer could also receive intramuscular fulvestrant 500 mg on day 1 and day 15 of cycle 1, and day 1 of each 28-day cycle thereafter; other anticancer agents (except luteinizing hormone-releasing hormone [LHRH] analogs for patients with breast or prostate cancer) and radiotherapy were avoided.

Patients who completed part A without disease progression or unacceptable toxicity, and who were considered by the investigator as likely to benefit from further capivasertib treatment, entered part B (treatment period 4) of the study, in which the efficacy of capivasertib (with or without standard-of-care anticancer treatment, fulvestrant, paclitaxel, docetaxel, abiraterone, or olaparib), was evaluated as an exploratory endpoint. Patients continued to receive capivasertib 400 mg BID dosing on an intermittent schedule of 4 days on/3 days off (per 28-day cycle) until disease progression, unacceptable toxicity, withdrawal of consent, or any other reason for discontinuation of treatment (Fig. 1).

The study was conducted in accordance with the Declaration of Helsinki, the International Council for Harmonisation Good Clinical Practice guideline, and all applicable local regulations. The institutional review boards or independent ethics committees of all investigational sites approved the protocol, and all patients provided written consent.

### Study population

Adult patients (≥ 18 years of age) with body mass index (BMI) 18–32 kg/m^2^, and with documented evidence of locally advanced inoperable or metastatic solid tumors suitable for capivasertib treatment, were included in the study. Documented tumor alterations in PI3K/AKT pathway genes (by local testing) were recorded where available, but were not required for study inclusion after a protocol amendment (31 March 2022). Patients were required to have an Eastern Cooperative Oncology Group (ECOG)/World Health Organization (WHO) performance status (PS) of 0 or 1, with no deterioration over the previous 2 weeks, a minimum life expectancy of 12 weeks, and at least one lesion (measurable and/or non-measurable) that was accurately assessed at baseline by computed tomography (CT)/magnetic resonance imaging (MRI), or X-ray and was suitable for repeated assessment. Female patients were not lactating, not pregnant, and were not of childbearing potential (or, if they were of childbearing potential, had to abstain from sexual activity or use two forms highly effective contraceptive methods from screening until 4 weeks after discontinuation of study drug). Male patients had to abstain from sexual activity or use barrier contraceptive methods from screening until 16 weeks after discontinuation of study drug.

Exclusion criteria included: previous allogeneic bone marrow or solid organ transplant, major surgery, or radiotherapy with a wide field of radiation within 4 weeks prior to first dose of capivasertib; radiotherapy with a limited field of radiation for palliation within 2 weeks prior to study intervention initiation; unresolved toxicity from prior therapies of grade > 2; and any unresolved toxicity that may interfere with PK assessment. Patients with inadequate bone marrow reserve or organ function, including renal impairment (creatinine > 1.5 × upper limit of normal, concurrent with creatinine clearance < 50 mL/min), patients with cardiac ejection fraction < 50% or outside the institutional range of normal, and patients with clinically significant abnormalities in glucose metabolism (insulin treatment needed or glycated hemoglobin (HbA1c)  > 7.5%) or blood lipid profiles, were excluded; patients with alanine aminotransferase and aspartate transaminase > 2.5 × upper limit of normal, if no demonstrable liver metastases, or > 5 × upper limit of normal in the presence of liver metastases, as well as patients with total bilirubin > 1.5 × upper limit of normal were also excluded. Elevated alkaline phosphatase was not an exclusion criterion if, due to the presence of bone metastasis and liver function, it was otherwise considered adequate. Those with spinal cord compression or symptomatic brain metastases, or severe or uncontrolled systemic diseases (including uncontrolled hypertension, active bleeding diatheses, or clinically significant active infections) were also excluded.

Patients could not have received potent or moderate inhibitors or inducers of CYP3A4 within the 2 weeks (or 5 half-lives, whichever was longer) prior to the first dose of study intervention (3 weeks for St. John’s wort, and 4 weeks for enzalutamide), and could not have received sensitive substrates of CYP3A4 with a narrow therapeutic window within 1 week prior to the first dose of study intervention. Patients also could not have received any investigational agent from a previous study within 30 days of the first dose of study drug, or any chemotherapy, immunotherapy, immunosuppressant medication (other than corticosteroids), or anticancer agents (except LHRH analogs in patients with breast or prostate cancer) within 2 weeks (or 5 half-lives, whichever was longer) of the first dose of study intervention. Nitrosourea or mitomycin C within 6 weeks of the first dose of study intervention was not permitted.

### Drug administration and restrictions

In part A, midazolam (and thus capivasertib, when given concomitantly) was given in the morning after an overnight fast to minimize PK variability; on the other days, capivasertib was dosed in a fasted state from at least 2 h prior to the dose to at least 1 h post-dose, where possible. In part B, capivasertib was given with or without food at the investigator’s discretion.

Dose modifications for midazolam were not permitted. A maximum of two dose reductions for capivasertib were permitted on the intermittent schedule (4 days on/3 days off), the first dose reduction from 400 to 320 mg BID and the second from 320 to 200 mg BID; dose re-escalations were not allowed. Substantial acute toxicities (any-grade intolerable AEs, grade 1 or 2 clinically significant, intolerable AEs, or grade ≥ 3 AEs related to capivasertib) were managed as medically indicated, including with temporary suspension of capivasertib.

Concomitant administration of drugs known to prolong the QT interval was restricted, or patients were closely monitored if such drugs were considered essential to patient management. Substrates for CYP3A4, multidrug and toxin extrusion 1 (MATE1), or organic cation transporter 2 (OCT2) transport were either avoided or used cautiously, and statins were avoided or used at capped doses. In addition to the prior/concomitant therapies documented in the exclusion criteria, patients avoided herbal supplements (e.g., St. John’s wort) and the ingestion of foods and beverages known to modulate CYP3A4 during the study; grapefruit juice and Seville oranges (known CYP3A4 inhibitors) were prohibited from 7 days prior to initiating treatment period 1 until after the completion of PK sample collection in part A.

Additional restrictions were implemented in part A of the study: other anticancer agents (except LHRH analogs for patients with breast or prostate cancer, and fulvestrant) and radiotherapy were avoided, although radiation for palliation at focal sites was permitted. The use of midazolam was prohibited from at least 14 days before the first on-study administration of midazolam until the collection of the last PK sample for either treatment.

### Sample collection and PK assessments

To assess the full PK profile of midazolam, 2 mL blood samples were collected at 0 (pre-dose), 0.25, 0.5, 0.75, 1, 1.5, 2, 3, 4, 6, 8, and 12 h post-midazolam dose on days 1, 8, and 12 of cycle 1, corresponding to midazolam alone on day 1 (treatment period 1), midazolam without capivasertib on day 8 (3rd day off capivasertib; treatment period 3), and midazolam with capivasertib on day 12 (4th day on capivasertib; treatment period 3). Single 2-mL 24-h post-dose samples were also collected on days 2, 9, and 13 of the same cycle.

Blood was sampled for capivasertib PK analysis on day 9 (one pre-capivasertib morning dose sample), day 12 (full PK profile: 0 [pre-capivasertib morning dose], 0.5, 1, 2, 4, 6, 8, and 12 h post-capivasertib morning dose), and day 13 (one 12-h post-day 12 capivasertib evening sample) of cycle 1, again by obtaining 2-mL blood samples.

Study samples were received frozen with dry ice, and stored at –60 to –80 °C. Analysis of plasma concentrations of midazolam was performed by Labcorp Early Development Laboratories Inc. using a liquid chromatography–mass spectrometry method that was fully validated according to US Food and Drug Administration bioanalytical guidelines. The samples were extracted using supported liquid extraction (SLE). Specifically, 50 µL of plasma was diluted with 25 µL of the internal standard (midazolam-d4) and 200 µL water: ammonium hydroxide (95:5). The diluted sample (200 µL) was loaded onto a Biotage Isolute^®^ SLE + cartridge 200 µL and subsequently eluted with 1000 µL of methyl tert-butyl ether. The resulting extract was dried under nitrogen at 40 °C and then reconstituted in 150 µL of methanol: water: formic acid (50:50:0.1). Chromatographic separation was performed using a Shimadzu Prominence 20-series high-performance liquid chromatography system equipped with a Mac-Mod ACE C18 column (50 × 2.1 mm dimensions, 5 µm). The liquid chromatography separations were carried out at 30°C with a flow rate of 0.600 mL/min. Initially, a 0.25-min hold at 50% Mobile Phase B was maintained, followed by a linear gradient from 50 to 95% B over 1.25 min. Mass spectrometry detection was performed using a Sciex API 5500™ triple quadrupole tandem mass spectrometry system operating in positive mode. The following multiple reaction monitoring transitions were utilized: midazolam (326.3 → 291.3) and internal standard (330.3 → 295.1). Quantitation was based on linear regression with a 1/x^2 weighting factor. The method’s validated concentration range was 0.0500–50.0 ng/mL midazolam with intra-batch variation of ±4.5% bias and ±6.3% relative standard deviation (RSD) and inter-batch variation of ±3.0% bias and ±5.2% RSD observed during validation. The results of an incurred sample reproducibility assessment performed for midazolam samples during the sample analysis phase of the study, showed a relative percentage difference within ±20% for 98.8% of the samples assessed. Plasma concentrations of capivasertib were determined using high-performance liquid chromatography with tandem mass spectrometric detection, using validated analytical methods as described previously [12].

All PK parameters were determined from plasma concentrations using non-compartmental methods with Phoenix^®^ WinNonlin^®^ Version 8.3.5 (Certara, Princeton, NJ). PK parameters assessed for midazolam in the absence of capivasertib (day 1 of cycle 1; treatment period 1) and during capivasertib treatment on an intermittent 4 days on, 3 days off schedule (day 8 and day 12 of cycle 1; treatment period 3) included: maximum observed plasma (peak) drug concentration (C_max_); area under the concentration–time curve from time zero to infinity (AUC_inf_); area under the plasma concentration–time curve from zero to the last quantifiable concentration (AUC_last_); time to reach maximum observed plasma drug concentration (*t*_max_); and half-life associated with terminal slope of a semi-logarithmic concentration–time curve (*t*½_λz_).

PK parameters assessed for capivasertib included: *C*_max_, area under the plasma concentration–time curve in the dose interval (AUC_τ_), *t*½_λz_, and *t*_max_ of capivasertib and its glucuronide metabolite, apparent total body clearance of capivasertib from plasma after oral administration (CL/F), and trough plasma concentration (*C*_trough_) of capivasertib and its glucuronide metabolite on days 9 and 13 of cycle 1.

### Safety and exploratory efficacy analyses

Safety and tolerability were evaluated in terms of AEs, which were classified and graded according to the National Cancer Institute (NCI) Common Terminology Criteria for Adverse Events (CTCAE) v5.0; vital signs; 12-lead electrocardiogram (ECG); and clinical laboratory assessments, including clinical chemistry and hematology parameters, urinalysis, HbA1c levels, and lipid profiles. Patients were followed for safety for at least 30 days after the last dose of capivasertib.

Efficacy was evaluated as an exploratory endpoint by investigator assessment, and per Response Evaluation Criteria in Solid Tumors (RECIST) version 1.1. Tumor assessments were performed by CT and/or MRI scans at screening and then every 8 weeks (± 7 days; earlier if clinically indicated) for 2 years, and then every 12 weeks until disease progression. Radiographic bone scans were performed at screening and repeated as clinically indicated, depending on tumor type. Efficacy endpoints were: PFS, defined as time from start of capivasertib dosing (day 2 of cycle 1) until disease progression; objective response rate (ORR), defined as the proportion of patients with measurable disease at baseline who have a confirmed complete or partial response; duration of response (DoR), defined as time from the date of the first documented response until the date of documented progression or death in absence of progression; and clinical benefit rate (CBR) at 24 weeks, defined as the percentage of patients who have a confirmed complete or partial response, or who have stable disease for at least 23 weeks from the start of capivasertib dosing (day 2 of cycle 1).

### Statistical analyses

The number of patients included in the study was selected to gain sufficient precision for the 90% CI of the least squares (LS) geometric means ratio (GMR) of midazolam AUC_inf_ and *C*_max_, while exposing as few patients as possible to study drugs and procedures. Based on the estimates of the within-patient coefficient of variation (CV) of 0.3 for log AUC_inf_ and 0.35 for *C*_max_, a sample size of 14 evaluable patients was projected to provide relative precision (ratio between the upper and lower limits of the 90% CI) of 1.6 and 1.7 for AUC_inf_ and *C*_max_, respectively, with a probability of 80%.

Plasma midazolam or capivasertib PK parameters were analyzed for the PK analysis set, consisting of all patients who received at least one dose of midazolam and capivasertib and had at least one reportable post-dose plasma concentration. Patients were also required to have day 1 and day 8 or Day 12 PK data to be included in statistical analyses. Patients with protocol deviations, dose interruptions or reductions, or AEs that could have significantly impacted the PK of capivasertib and/or midazolam were considered non-evaluable.

Statistical analyses of *C*_max_ and AUC_inf_ were performed using a mixed-effects model following a natural logarithmic transformation of the PK parameters, with fixed effect for day and random effect for patient. LS geometric means of treatment effect and the corresponding two-sided 95% CIs, as well as ratios of LS geometric means together with two-sided 90% CIs of test treatment (corresponding to the 3rd day off or 4th day on capivasertib; day 8 or day 12 of cycle 1) and reference treatment (midazolam alone; day 1 of cycle 1), were estimated. AUC_inf_ or *C*_max_ ratios with 90% CIs between 0.80 and 1.25 indicated no interaction between midazolam and capivasertib. A ratio of LS geometric means of AUC_inf_ in the ranges of ≥ 1.25 to < 2, ≥ 2 to < 5, or ≥ 5 indicated a weak, moderate, or strong CYP3A4 inhibitor classification, respectively, and a ratio of LS geometric means of AUC_inf_ in the ranges of ≥ 0.5 to < 0.8, ≥ 0.2 to < 0.5, or < 0.2 indicated a weak, moderate, or strong CYP3A4 inducer classification, respectively. The remaining PK parameters were summarized using descriptive statistics.

Safety analyses were performed on the full analysis set, which consisted of all patients who received at least one dose of midazolam and/or capivasertib, irrespective of protocol adherence or continued participation in the study. Efficacy analyses were also performed on the full analysis set. Summary and/or descriptive statistics were used to analyze baseline demographic, safety, and efficacy data; PFS and DoR were estimated using Kaplan–Meier methodology.

All statistical computations were performed using SAS^®^ 9.4 (SAS Institute, Cary, NC). There was no imputation of missing data.

## Results

### Patient disposition

A total of 28 patients were enrolled in the study, of whom 21 received study treatment in part A and were included in the full analysis set and the PK analysis set (7 screening failures did not receive study treatment; see Online Resource 1: Supplementary Fig. 1). Of the 21 patients who received study treatment in part A, 20 completed part A and 19 went on to part B and received capivasertib monotherapy with or without standard-of-care anticancer treatment. There were four deaths during the study: one in part A (treatment period 2), and three in part B. All patients who died had discontinued capivasertib prior to death, because of clinical disease progression; none of the deaths were deemed by the investigator to be possibly related to study treatment.

Although permitted in part A, no patients with estrogen receptor-positive breast cancer received concomitant fulvestrant in the study. Fifteen patients were deemed to have had at least one important protocol deviation (Online Resource 1: Supplementary Table 1). Following exclusions due to important protocol deviations and the presence of quantifiable (> 0.05 ng/mL) pre-dose concentrations for midazolam in some PK profiles (prior to cycle 1, day 1 and at cycle 1, day 8 and day 12), a total of 18 patients were included in the PK statistical analysis.

### Patient demographics and baseline characteristics

The demographic and baseline characteristics of the 21 patients included in the full analysis set are summarized in Table 1. Most patients were female (66.7%), and the median (range) age was 65 years (38–77). Most patients were of White race (90.5% [not Hispanic/Latino 81%]) and had an ECOG PS of 0 (61.9%). Most patients entered the study with metastatic disease (85.7%), and the most common primary tumor sites were the colon (23.8%), uterus (19%), and lung (14.3%). Most patients (95.2%) had tumors harboring PI3K/AKT pathway alterations. Patients had received a median of 3 (range 1– 9) prior anticancer therapies.

**Table 1:** Demographics and baseline characteristics (full analysis set)

	Total (*N* = 21)
Age, median (range), years	65.0 (38–77)
Female, *n* (%)	14 (66.7)
Race, *n* (%)	
White	19 (90.5)
Black or African American	2 (9.5)
Ethnic group, *n* (%)	
Hispanic or Latino	4 (19.0)
Not Hispanic or Latino	17 (81.0)
Weight, median (range), kg	73.1 (51.1–101.3)
BMI, median (range), kg/m^2^	25.8 (20.5–31.3)
ECOG PS, *n* (%)	
0	13 (61.9)
1	5 (23.8)
Missing	3 (14.3)
Extent of disease upon entry, *n* (%)	
Metastatic	18 (85.7)
Locally advanced	0
Both metastatic and locally advanced	3 (14.3)
Primary tumor location, *n* (%)	
Colon	5 (23.8)
Uterus	4 (19.0)
Lung	3 (14.3)
Liver	1 (4.8)
Anal canal	1 (4.8)
Anus	1 (4.8)
Endometrium	1 (4.8)
Esophagus	1 (4.8)
Lymph node	1 (4.8)
Ovary	1 (4.8)
Peritoneum	1 (4.8)
Rectum	1 (4.8)
Any PI3K/AKT pathway alteration^a^, *n* (%)	20 (95.2)
* PIK3CA*	16 (76.2)
* AKT2*	1 (4.8)
* PTEN*	5 (23.8)
* PIK3R2*	1 (4.8)
Prior anticancer therapies, *n* (%)	20 (95.2)
Anthracyclines	4 (19.0)
Aromatase inhibitors	5 (23.8)
Fluorouracil, folinic acid, oxaliplatin combinations	5 (23.8)
Folinic acid	4 (19.0)
Monoclonal antibodies and antibody–drug conjugates	3 (14.3)
Protein kinase inhibitors	6 (28.6)
PD-1/PDL-1 inhibitors	9 (42.9)
Platinum compounds	13 (61.9)
Pyrimidine analogs	13 (61.9)
Taxanes	11 (52.4)
Topoisomerase I inhibitors	6 (28.6)
VEGF/VEGFR inhibitors	8 (38.1)

*AKT* AKT serine/threonine kinase, *BMI* body mass index, *ECOG PS* Eastern Cooperative Oncology Group performance status, *N* number of participants per treatment group, *n* number of patients per category, *PD-1* programmed cell death protein 1, *PD-L1* programmed cell death ligand 1, *PI3K* phosphoinositide 3-kinase, *PIK3CA*, phosphatidylinositol-4,5-bisphosphate 3-kinase catalytic subunit alpha; *PIK3R2*, phosphoinositide-3-kinase regulatory subunit 2; *PTEN *phosphatase and tensin homolog, *VEGF* vascular endothelial growth factor, *VEGFR* VEGF receptor

^a^One patient may have more than one gene alteration at the same time

### Exposure to treatment

Analysis was conducted with a data cut-off of 11 May 2023. In part A, the median total treatment duration was 11 (range 2–21) days, and the median actual treatment duration was 8 (range 2–9) days. The total number of midazolam doses administered over the course of the study was 58: 21 in treatment period 1 (midazolam only on day 1 of cycle 1) and 37 in treatment period 3 (midazolam administered on day 8 and day 12 of cycle 1). Two patients missed one dose of midazolam during treatment period 3, and one patient withdrew from the study prior to treatment period 3 (withdrawal due to death).

#### Pharmacokinetics

##### Effect of capivasertib on midazolam exposure

**Fig. 2:**
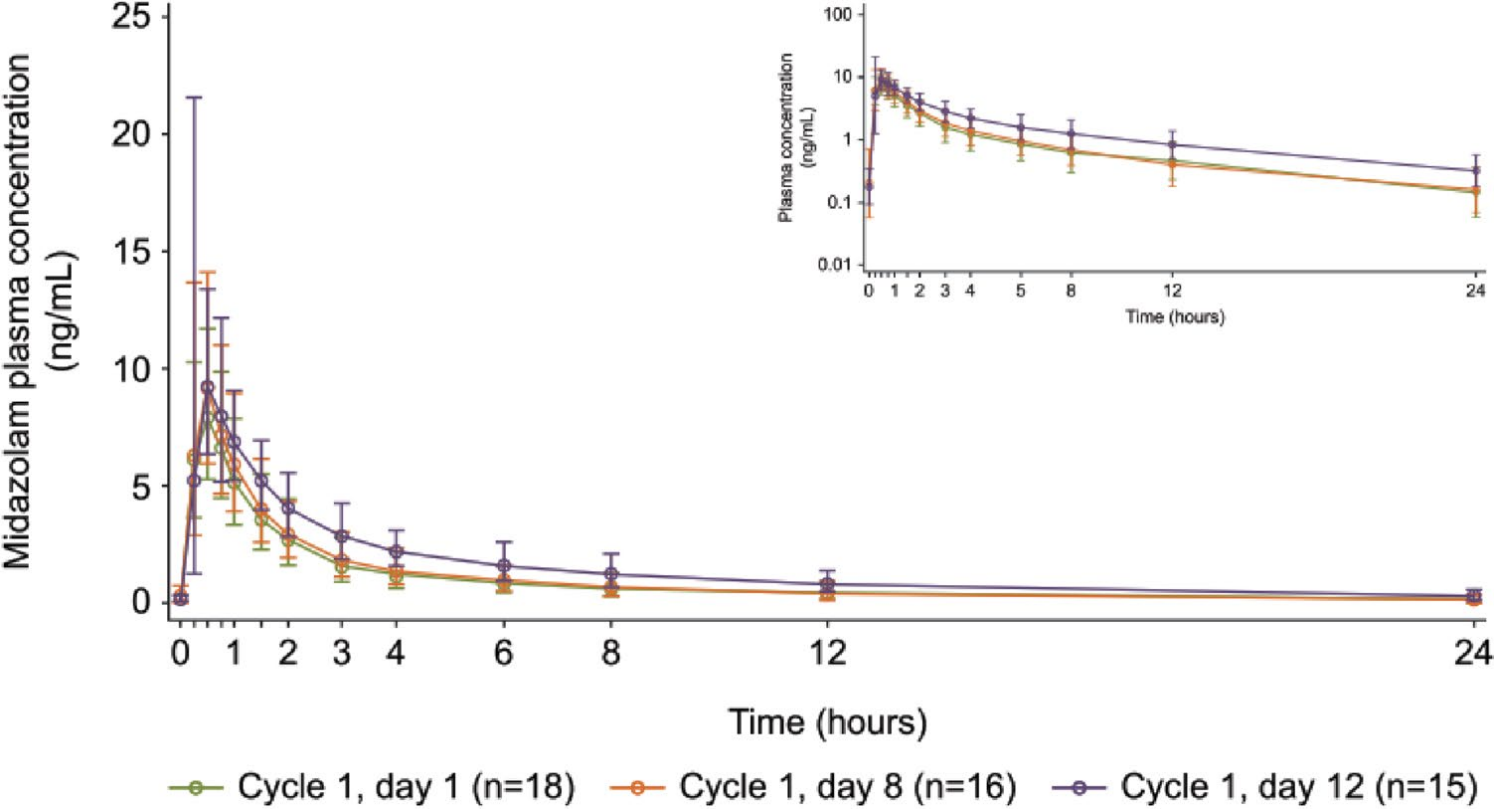
Midazolam plasma concentration over time in part A. Points and error bars show the geometric mean ± geometric SD of plasma concentrations. Inset shows geometric mean values plotted on a semi-logarithmic scale. Treatment period 1 (day 1 of cycle 1): patients received a single oral dose of midazolam (1 mg). Treatment period 3 (day 8 of cycle 1): patients received a single oral dose of midazolam (1 mg) on day 8, corresponding to the 3rd day off capivasertib (400 mg BID on an intermittent schedule [4 days on/3 days off]). Treatment period 3 (day 12 of cycle 1): patients received a single oral dose of midazolam (1 mg) on day 12, corresponding to the 4th day on capivasertib (400 mg BID on an intermittent schedule [4 days on/3 days off]). BID, twice daily; *n*, number of patients in the PK analysis set per treatment group; *PK* pharmacokinetic, *SD* standard deviation

Midazolam exposure increased when coadministered with capivasertib. The geometric mean midazolam plasma concentration versus time for day 1 of cycle 1 (midazolam alone) was similar to day 8 of cycle 1 (corresponding to the 3rd day off capivasertib) but remained higher for day 12 of cycle 1 (corresponding to the 4th day on capivasertib). Following *t*_max_, concentrations of midazolam decreased in a biphasic manner, with similar profile shapes for days 1, 8, and 12 (Fig. 2).

**Fig. 3:**
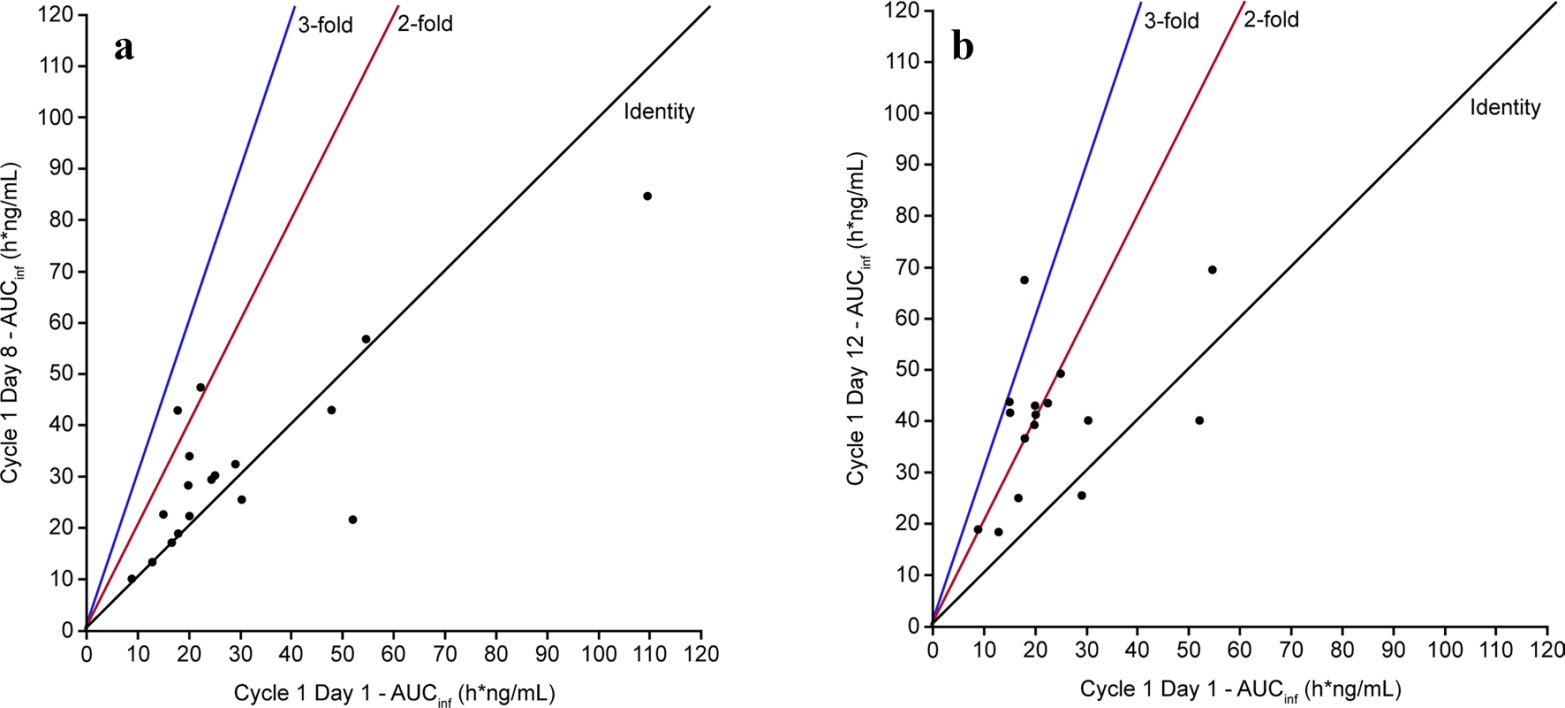
AUC_inf_ values for midazolam from each individual participant in the study for day 1 vs day 8 of cycle 1 (**a**) and day 1 vs day 12 of cycle 1 (**b**). Lines show a 1:1 (identity), 2:1 (two-fold) or 3:1 (three-fold) relationship. AUC_inf_, area under the concentration–time curve from time of administration to infinity

Coadministration of midazolam with capivasertib did not appear to affect time to maximum midazolam concentration; median *t*_max_ was approximately 0.5 h post-dose for days 1, 8, and 12 of cycle 1, with similar ranges observed (0.3–0.9 h, 0.2–0.8 h, and 0.3–1.1 h post-dose, respectively). Estimates of *t*_½λz_ were also similar with geometric mean values of 7.24, 8.52, and 7.23 h for days 1, 8, and 12 of cycle 1, respectively. Midazolam AUC_last_ was higher for day 12 of cycle 1 than for day 8 of cycle 1 at 34.70 h*ng/mL versus 24.49 h*ng/mL; both were higher than the AUC_last_ for day 1 of cycle 1 (midazolam alone) at 22.39 h*ng/mL. The summary statistics for these and other midazolam PK parameters with and without capivasertib are shown in Table 2. The inter-patient variability (geometric CV) in AUC_inf_ was moderate and numerically lower when midazolam was given after pre-treatment with capivasertib (day 8, cycle 1 and day 12, cycle 1), compared to when midazolam was given alone (day 1, cycle 1). On day 8, cycle 1, most of the individual patient’s midazolam AUC_inf_ values were close to the values on day 1, cycle 1 (Fig. 3A), and on day 12, cycle 1, most of the values were two-fold higher than on day 1, cycle 1 (Fig. 3B), with few exceptions in both directions.

**Table 2:** PK parameters of midazolam with or without capivasertib in part A (PK analysis set)

Parameter	Summary statistics	Treatment period 1	Treatment period 3		GMR (90% CI)	
		Day 1 of cycle 1 (midazolam alone; *N* = 18)^a^	Day 8 of cycle 1 (midazolam without capivasertib; *N* = 16)^b^	Day 12 of cycle 1 (midazolam with capivasertib; *N* = 15)^c^	Day 8 vs. day 1 of cycle 1	Day 12 vs. day 1 of cycle 1
AUC_inf_ (h*ng/mL)	Geometric mean (gCV%)	25.02^a^ (65.1)	28.28^b^ (53.6)	39.55^c^ (35.3)	1.13 (0.97–1.32)	1.75 (1.50–2.05)
AUC_last_ (h*ng/mL)	Geometric mean (gCV%)	22.39^a^ (52.0)	24.49^b^ (45.3)	34.70^c^ (32.5)		
*C*_max_ (ng/mL)	Geometric mean (gCV%)	8.55^a^ (39.6)	9.90^b^ (53.4)	9.83^c^ (39.6)	1.15 (0.95–1.33)	1.25 (1.08–1.46)
*t*_max_ (h)	Median (range)	0.53^a^ (0.3–0.9)	0.46^b^ (0.2–0.8)	0.55^c^ (0.3–1.1)		
*t*_½λz_ (h)	Geometric mean (gCV%)	7.24^a^ (40.3)	8.52^b^ (65.2)	7.23^c^ (38.3)		

Treatment period 1 (day 1 of cycle 1): patients received a single oral dose of midazolam (1 mg). Treatment period 3 (day 8 of cycle 1): patients received a single oral dose of midazolam (1 mg) on day 8, corresponding to the 3rd day off capivasertib (400 mg BID on an intermittent schedule [4 days on/3 days off]). Treatment period 3 (day 12 of cycle 1): patients received a single oral dose of midazolam (1 mg) on day 12, corresponding to the 4th day on capivasertib (400 mg BID on an intermittent schedule [4 days on/3 days off])

*AUC*_*inf*_ area under the plasma concentration–time curve from zero to infinity, *AUC*_*last*_ area under the plasma concentration–time curve from zero to last observed timepoint, *BID* twice daily, *CI* confidence interval, *C*_*max*_ maximum observed plasma (peak) drug concentration, *gCV%* geometric coefficient of variation, *GMR* geometric mean ratio, *N* number of participants included in analysis, *n* number of participants who received the dose, *PK* pharmacokinetics, *t*_*½λz*_ half-life associated with terminal slope (λz) of a semi-logarithmic concentration–time curve, *t*_*max*_ time to reach maximum observed plasma (peak) drug concentration

^*a*^*n* = *21 patients were dosed (selected patients with protocol deviations were excluded)*

^*b*^*n* = *20 patients were dosed (selected patients with protocol deviations were excluded)*

^*c*^*n* = *17 patients were dosed (selected patients with protocol deviations were excluded)*

Coadministration of midazolam with capivasertib increased midazolam exposure more than 1.25-fold but less than two-fold on day 12. The GMR (90% CI) of AUC_inf_ for day 12 versus day 1 of cycle 1 was 1.75 (1.50–2.05). Peak exposure also increased, with a GMR (90% CI) of 1.25 (1.08–1.46) for *C*_max_. A small numerical increase in midazolam exposure was observed for day 8 versus day 1 of cycle 1, with a GMR (90% CI) of 1.13 (0.97–1.32) for AUC_inf_ and 1.15 (0.95–1.33) for *C*_max_ (Table 2). Taken together, these data suggest that capivasertib is a weak inhibitor of CYP3A4.

##### PK profile of capivasertib and its glucuronide metabolite

Plasma concentrations of capivasertib and AZ14102143, its glucuronide metabolite, were quantifiable from pre-dose to 12 h post-dose for all patients on day 12 of cycle 1 (corresponding to the 4^th^ day on capivasertib, and in the presence of a single oral dose of midazolam) (see Online Resource 1: Supplementary Fig. 2). Following *t*_max_, concentrations for capivasertib and AZ14102143 decreased with similar profile shapes for both analytes.

A peak capivasertib concentration of 1779 ng/mL was reached at 1.5 h post-dose, and a peak AZ14102143 concentration of 11,220 ng/mL was reached at 2.0 h post-dose. The AUC and t½ over the 12-h dosing interval was 8499 h*ng/mL and 3.76 h for capivasertib, and 68,810 h*ng/mL and 3.40 h for AZ14102143. *C*_trough_ on day 13 of cycle 1 was 281.5 ng/mL for capivasertib and 2575 ng/mL for AZ14102143, and on day 9 of cycle 1 (corresponding to 3 off-capivasertib-dosing days) was 10.96 ng/mL for capivasertib and 45.22 ng/mL for AZ14102143. The apparent clearance of capivasertib was 46.4 L/h. Moderate inter-patient variability was observed, as reflected by the geometric CV for *C*_max_ and AUC parameters. The summary statistics for capivasertib and AZ14102143 PK parameters are shown in Online Resource 1: Supplementary Table 2.

### Safety

The median total capivasertib treatment duration across part A and part B was 60.0 (range 3–228) days, and the median actual capivasertib treatment duration was 31.0 (range 3–127) days. All patients experienced at least one AE during the study. The most frequently reported AEs by treatment period are shown in Table 3. A lower incidence of AEs occurred during treatment period 1 (single midazolam dose) compared with treatment periods 2 (capivasertib alone) and 3 (capivasertib + midazolam). The most frequently reported AEs (frequency > 25% of patients overall) included diarrhea (76.2%), nausea and fatigue (both 33.3%), and hyperglycemia and anemia (both 28.6%).

**Table 3:** Summary of AEs per treatment period (any AE in ≥ 15% of patients in any treatment period, and all grade ≥ 3 AEs; full analysis set)

AE, *n* (%)	Part A						Part B		Total (*N* = 21)	
	Treatment period 1: midazolam only (*N* = 21)		Treatment period 2: capivasertib only (*N* = 21)		Treatment period 3: capivasertib + midazolam (*N* = 20)		Treatment period 4: capivasertib only (*N* = 19)			
Grade	Any	Grade ≥ 3	Any	Grade ≥ 3	Any	Grade ≥ 3	Any	Grade ≥ 3	Any	Grade ≥ 3
Any AE	3 (14.3)	0	10 (47.6)	3 (14.3)	16 (80.0)	6 (30.0)	17 (89.5)	9 (47.4)	21 (100)	14 (66.7)
Diarrhea	0	0	6 (28.6)	0	5 (25.0)	0	7 (36.8)	1 (5.3)	16 (76.2)	1 (4.8)
Rash (group term)^a^	0	0	0	0	6 (30.0)	3 (15.0)	2 (10.5)	0	8 (38.1)	3 (14.3)
Nausea	0	0	2 (9.5)	0	2 (10.0)	0	4 (21.1)	0	7 (33.3)	0
Fatigue	0	0	0	0	3 (15.0)	0	4 (21.1)	2 (10.5)	7 (33.3)	2 (9.5)
Anemia	0	0	2 (9.5)	1 (4.8)	0	0	4 (21.1)	0	6 (28.6)	1 (4.8)
Hyperglycemia	1 (4.8)	0	1 (4.8)	0	3 (15.0)	1 (5.0)	3 (15.8)	0	6 (28.6)	1 (4.8)
Decreased appetite	0	0	0	0	1 (5.0)	0	4 (21.1)	2 (10.5)	5 (23.8)	2 (9.5)
Hypokalemia	0	0	1 (4.8)	0	2 (10.0)	0	3 (15.8)	1 (5.3)	5 (23.8)	1 (4.8)
Hyponatremia	0	0	1 (4.8)	0	0	0	3 (15.8)	1 (5.3)	4 (19.0)	1 (4.8)
Hypotension	0	0	0	0	1 (5.0)	0	3 (15.8)	1 (5.3)	4 (19.0)	1 (4.8)
Blood creatinine increased	0	0	0	0	0	0	3 (15.8)	0	3 (14.3)	0
Asthenia	0	0	1 (4.8)	0	0	0	2 (10.5)	1 (5.3)	3 (14.3)	1 (4.8)
Lymphocyte count decreased	0	0	1 (4.8)	1 (4.8)	0	0	2 (10.5)	0	3 (14.3)	1 (4.8)
Abdominal pain	0	0	1 (4.8)	0	0	0	1 (5.3)	1 (5.3)	2 (9.5)	1 (4.8)
COVID-19	0	0	0	0	1 (5.0)	0	1 (5.3)	1 (5.3)	2 (9.5)	1 (4.8)
Blood bilirubin increased	0	0	0	0	0	0	2 (10.5)	1 (5.3)	2 (9.5)	1 (4.8)
Thrombocytopenia	0	0	1 (4.8)	1 (4.8)	0	0	0	0	1 (4.8)	1 (4.8)
Large intestinal obstruction	0	0	0	0	0	0	1 (5.3)	1 (5.3)	1 (4.8)	1 (4.8)
Blood alkaline phosphatase increased	0	0	0	0	1 (5.0)	1 (5.0)	0	0	1 (4.8)	1 (4.8)
Gamma-glutamyl transferase increased	0	0	0	0	0	0	1 (5.3)	1 (5.3)	1 (4.8)	1 (4.8)
Hypoalbuminemia	0	0	1 (4.8)	1 (4.8)	0	0	0	0	1 (4.8)	1 (4.8)
Ureteric obstruction	0	0	0	0	0	0	1 (5.3)	1 (5.3)	1 (4.8)	1 (4.8)
Urticaria	0	0	0	0	1 (5.0)	1 (5.0)	0	0	1 (4.8)	1 (4.8)
Sepsis	0	0	0	0	0	0	1 (5.3)	1 (5.3)	1 (4.8)	1 (4.8)

Treatment period 1 (day 1 of cycle 1): patients received a single oral dose of midazolam (1 mg). Treatment period 2 (days 2–7 of cycle 1): patients received repeated oral doses of capivasertib 400 mg BID, given as an intermittent schedule (4 days on/3 days off). Treatment period 3 (days 8–15 of cycle 1): patients received single oral doses of midazolam (1 mg) on days 8 and 12 during intermittent capivasertib treatment (400 mg BID, 4 days on/3 days off). Treatment period 4 (day 16 of cycle 1 onwards): patients received repeated oral doses of capivasertib 400 mg BID, given as an intermittent schedule (4 days on/3 days off)

*AE* adverse event, *BID* twice daily, *COVID-19* coronavirus disease 2019, *N* number of participants, *n* number of observations in analysis

^a^The group term of rash includes the Preferred Terms of rash, rash macular, maculopapular rash, rash papular and rash pruritic

In total, 14 (66.7%) patients experienced grade ≥ 3 AEs. During part A, the rate of grade ≥ 3 AEs was higher in treatment period 3 (capivasertib) than in treatment periods 1 or 2 (Table 3). Twelve patients (57.1%) experienced grade 3 AEs; only rash (group term *n* = 3; 14.3%) and fatigue and decreased appetite (both *n* = 2; 9.5%) were reported in more than one patient (in part B). One patient (4.8%) experienced a grade 4 AE (thrombocytopenia) during treatment period 2; this was not considered related to either capivasertib or midazolam. One patient (4.8%) experienced a grade 5 AE (sepsis) in part B; the death was not considered related to either capivasertib or midazolam. A total of five serious AEs (SAEs) were reported in the study (*n* = 1 in treatment period 2, *n* = 1 in treatment period 3, and *n* = 3 in part B). Only one SAE (treatment period 3) was considered related to study treatment (rash considered related to both capivasertib and midazolam).

Five patients (23.8%) required a capivasertib dose reduction due to AEs: *n* = 1 in treatment period 3 and *n* = 4 in part B. Fourteen patients (66.7%) required a capivasertib dose interruption due to AEs (*n* = 6 patients in treatment period 3, *n* = 7 patients in part B, and *n* = 1 patient with an event in treatment period 3 and part B). Three patients experienced AEs leading to permanent discontinuation of capivasertib. Only one patient discontinued capivasertib because of an AE considered related to capivasertib and midazolam (fatigue during part B).

Although some fluctuations or other abnormalities were observed in laboratory results, vital signs, physical examinations, or ECG during part A of the study, these were expected, given the advanced nature of the patients’ disease and previous clinical experience with capivasertib and midazolam. There were no reports of QT prolongation in the study.

### Exploratory analysis of efficacy

**Fig. 4:**
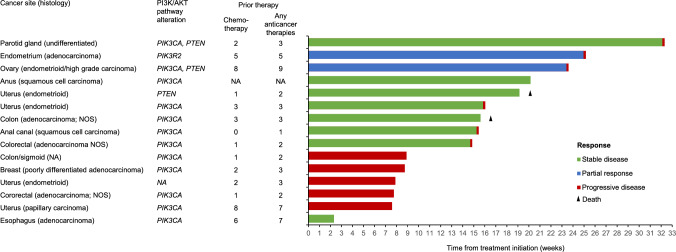
Time on treatment in patients evaluable for response (*n* = 15). *AKT *serine/threonine kinase, *NA* not available, *NOS* not otherwise specified, *PI3K* phosphoinositide 3-kinase, *PTEN* phosphatase and tensin homolog, *RECIST 1.1* Response Evaluation Criteria in Solid Tumors version 1.1

There was a total of 15 PFS events at the time of analysis, and median PFS (95% CI) was 3.4 months (1.8–5.4). Fifteen patients had measurable disease at baseline and at least one post-baseline scan; response to treatment over time for these patients is shown in Fig. 4. The ORR (95% CI) was 9.5% (1.2–30.4). Two patients had a confirmed partial response to treatment: one with endometrial adenocarcinoma and phosphoinositide-3-kinase regulatory subunit 2 (*PIK3R2*)  alteration, and one with ovarian carcinoma and *PIK3CA* and *PTEN* alterations. The CBR (95% CI) at 24 weeks was 14.3% (3.1–36.3); three patients met the criteria for clinical benefit. Median DoR (95% CI) was 3.8 months (3.6–not calculable).

## Discussion

The primary aim of this phase 1, open-label, fixed-sequence study was to examine the effect of capivasertib on the activity of CYP3A, a key enzyme involved in the metabolism and clearance of numerous drugs. To this end, we evaluated the PK of midazolam, when administered alone and in combination with repeated doses of capivasertib, in patients with a broad range of metastatic solid tumors and prior anticancer therapies. Midazolam is a well-established sensitive substrate of CYP3A and is often used both in vitro and in vivo as a probe for potential inhibitors of this cytochrome P450 [13]. The LS GMR of 1.75 for midazolam AUC_inf_ on the 4th day on capivasertib (day 12 of cycle 1) was within the defined range for a weak inhibitor of CYP3A (≥ 1.25 and < 2.0) [11], indicating that capivasertib is a weak inhibitor of CYP3A when administered at a dose of 400 mg BID on an intermittent schedule (4 days on/3 days off). There was a small numerical increase in the midazolam AUC on the 3rd off-dosing day (LS GMR 1.13). However, as the CI included 1.00, no definite conclusion on capivasertib inhibition of CYP3A4 on the off-dosing day could be drawn. It should be noted, however, that the maximum inhibition of CYP3A4 is expected on the 4th dosing day of capivasertib each week at steady state and that the average inhibition over a cycle is less than the inhibition observed on day 12. These results confirm previous evidence from preclinical in vitro studies suggesting that capivasertib is an inhibitor of CYP3A. The exposure of the glucuronide metabolite of capivasertib was significantly higher than for parent capivasertib, but the half-lives were similar. This metabolite produced little or no CYP inhibition in vitro (unpublished data), but it should be noted that the drug–drug interaction observed in this study includes potential effects of this and other metabolites at the clinically relevant dosage.

As many anticancer agents (and other drugs administered for conditions that frequently occur in patients with advanced cancer) are CYP3A4 substrates [11], capivasertib is likely to be administered concomitantly with CYP3A substrates in clinical practice. The finding that capivasertib weakly inhibits CYP3A enzymes suggests that concomitant administration with drugs that are CYP3A substrates may lead to some temporary increases in their exposure, potentially leading to enhanced toxicity if they have a narrow therapeutic window. In such cases, close patient monitoring and/or small dose modifications and/or treatment modifications may be necessary. Although likely a rare occurrence, it should also be considered that the weak inhibition of CYP3A enzymes by capivasertib has the potential to reduce the efficacy of prodrugs activated by these enzymes.

In this study, pre-dose samples from seven patients displayed unexpected quantifiable concentrations of midazolam in both periods 1 and 3. For these patients, exclusions were applied to either the individual pre-dose concentrations and/or the whole affected PK profile, based on the concentration relative to the subsequent *C*_max._ Therefore, this is not considered to have affected the derived midazolam PK parameters included in the statistical analysis. The resulting dataset was sufficient to draw reliable conclusions on the drug–drug interaction between midazolam and capivasertib. The PK profiles of capivasertib and its glucuronide metabolite (day 12 of cycle 1) were in line with those observed in previous studies of capivasertib [3, 5, 14, 15]. As in other studies of capivasertib, moderate inter-patient variability was observed.

In this heavily pre-treated population of patients with advanced solid tumors, capivasertib was associated with a manageable safety profile, regardless of whether it was administered in the presence or absence of midazolam. In part A, most AEs were low grade and manageable by dose modifications or treatment, as per management guidance. In part B (capivasertib monotherapy), there was one grade 3 diarrhea event and no grade ≥ 3 rash or hyperglycemia events, suggesting capivasertib has a manageable tolerability profile in heavily pretreated patients with advanced stage disease. Only one treatment-related AE (fatigue), occurring during part B, led to a permanent discontinuation of capivasertib; the safety profile of capivasertib was broadly consistent with previous studies [6, 8–10]. Fluctuations in clinical chemistry, vital signs, and ECG parameters were as expected for a population with advanced cancer, and are in line with the known pharmacologic mode of action and previous clinical experience for capivasertib and midazolam; for example, midazolam is known to have a blood pressure-lowering effect [16]. Four patient deaths occurred during the study; none were deemed to be possibly related to study treatment.

Most patients were able to proceed to part B of the study. Exploratory analysis of efficacy indicated evidence of clinical activity as monotherapy in this heavily pre-treated population who had received up to nine prior anticancer therapies, the majority of whom had alterations of genes in the PI3K/AKT pathway. This included confirmed partial responses in two patients: one with endometrial adenocarcinoma harboring a *PIK3R2* alteration, and one with ovarian carcinoma harboring a *PIK3CA* and *PTEN* alterations.

## Conclusions

Capivasertib is a weak inhibitor of the CYP3A metabolic enzyme; therefore, the risk of clinically relevant drug–drug interactions caused by CYP3A4 inhibition is low. Findings from this study have informed recommendations for administering capivasertib in combination with drugs that are CYP3A substrates in future clinical trials, and in clinical practice. Capivasertib has a consistent and manageable safety profile at the therapeutic dose and schedule being pursued in clinical development, regardless of whether it is administered with midazolam. The exploratory analysis of efficacy indicates evidence of clinical activity as monotherapy in heavily pre-treated patients with advanced solid tumors harboring PI3K/AKT pathway alterations, as previously seen in other studies.

## Supplementary Information

Below is the link to the electronic supplementary material.

Supplementary file1 (DOCX 247 KB)

## Data Availability

Data underlying the findings described in this manuscript may be obtained in accordance with AstraZeneca’s data-sharing policy, described at: https://astrazenecagrouptrials.pharmacm.com/ST/Submission/Disclosure.
