# Supplementary material for: Pharmacokinetic study of capivasertib and the CYP3A4 substrate midazolam in patients with advanced solid tumors
Source: Cancer Chemother Pharmacol. 2024 Apr 20;94(2):223–35. doi: 10.1007/s00280-024-04667-3 (PMC11390765; doi:10.1007/s00280-024-04667-3)
Supplement: Supplementary file 1 — Supplementary file1 (DOCX 247 KB) [file 280_2024_4667_MOESM1_ESM.docx]

## Online Resource 1

## Supplementary Information

Pharmacokinetic study of capivasertib and the CYP3A4 substrate midazolam in patients with advanced solid tumors

*Cancer Chemotherapy and Pharmacology*

Claire Miller, Roberto Sommavilla, Cindy L. O’Bryant, Minal Barve, Afshin Dowlati, Jason J. Luke, Mahmuda Khatun, Thomas Morris, Marie Cullberg

**Supplementary Table 1** Important protocol deviations (part A; full analysis set)

|  | Treatment period 1 | Treatment period 2 | Treatment period 3 |  |
| --- | --- | --- | --- | --- |
|  | Midazolam only (*N*=21) | Capivasertib only (*N*=21) | Capivasertib + midazolam (*N*=20) | Total (*N*=21) |
| Patients with at least one important protocol deviation, *n* (%) | 7 (33.3) | 3 (14.3) | 6 (30.0) | 15 (71.4) |
| Pre-dose cycle 1, day 1 sample had midazolam present, *n* (%) | 7 (33.3) | 0 | 0 | 7 (33.3) |
| Received capivasertib on an off‑day, *n* (%)^a^ | 0 | 1 (4.8) | 3 (15.0)^~~a~~^ | 4 (19.0) |
| Documented use of disallowed medication, *n* (%)^b^ | 0 | 2 (9.5) | 0 | 2 (9.5) |
| Visit schedule not performed per study protocol, *n* (%) | 0 | 0 | 5 (25.0) | 5 (23.8) |
| Midazolam administration not performed at visit, *n* (%) | 0 | 0 | 1 (5.) | 1 (4.8) |
| Capivasertib PK sample not performed at visit, *n* (%) | 0 | 0 | 1 (5.0) | 1 (4.8) |
| Midazolam PK sample not performed at visit, *n* (%) | 0 | 0 | 1 (5.0) | 1 (4.8) |

One patient had an important protocol deviation in treatment period 1 and treatment period 3, but is counted only once in the total. The same patient may have more than one important protocol deviation. Treatment period 1 (day 1 of cycle 1): patients received a single oral dose of midazolam (1 mg). Treatment period 2 (days 2–8 of cycle 1): patients received repeated oral doses of capivasertib 400 mg BID, given as an intermittent schedule (4 days on/3 days off). Treatment period 3 (days 8–15 of cycle 1): patients received single oral doses of midazolam (1 mg) on days 8 and 12 during intermittent capivasertib treatment (400 mg BID, 4 days on/3 days off). Treatment period 4 (day 16 of cycle 1 onwards): patients received repeated oral doses of capivasertib 400 mg BID, given as an intermittent schedule (4 days on/3 days off)
AE, adverse event; BID, twice daily; *N*, number of participants; *n*, number of observations in analysis; PK, pharmacokinetic
^a^One patient was given capivasertib 400 mg in the morning of cycle 1, day 6 (a non-dosing day), two patients took capivasertib 400 mg in the morning of cycle 1, day 13 (a non-dosing day), and one patient incorrectly took capivasertib 400 mg in the evening of day 7 (the last non-dosing day of that week). No adjustments for these incorrect doses were made and none of these doses were associated with symptoms or AEs; ^b^One patient received cyclosporin and one patient received an investigational agent (monoclonal antibody)

**Supplementary Table 2** PK parameters of capivasertib and its metabolite, AZ14102143, on day 12 of cycle 1 (PK analysis set)

| Parameter | Summary  statistics | Capivasertib (*N*=17) | AZ14102143 (*N*=17) |
| --- | --- | --- | --- |
| C_max_ (ng/mL) | Geometric mean (gCV%) | 1779^a^ (48.3) | 11,220^a^ (42.7) |
| AUCτ (h*ng/mL) | Geometric mean (gCV%) | 8499^b^ (36.7) | 68,810^b^ (50.4) |
| t_max_ (h) | Median (range) | 1.48^a^ (0.6–5.5) | 2.03^a^ (0.6–7.6) |
| t_½λz_ (h) | Geometric mean (gCV%) | 3.76^c^ (43.7) | 3.40^d^ (62.4) |
| CL/F (L/h) | Geometric mean (gCV%) | 46.37^b^ (39.1) | NA |
| C_trough_ cycle 1, day 9 (ng/mL) | Geometric mean (gCV%) | 10.96^e^ (137.0) | 45.22^e^ (242.1) |
| C_trough_ cycle 1, day 13 (ng/mL) | Geometric mean (gCV%) | 281.5^f^ (53.3) | 2575^f^ (106.9) |

Treatment period 3 (day 12 of cycle 1): patients received a single oral dose of midazolam (1 mg) on day 12, corresponding to the 4^th^ day on capivasertib (400 mg BID on an intermittent schedule [4 days on/3 days off])
AUCτ, area under plasma concentration–time curve in the dose interval; BID, twice daily; CL/F, apparent total body clearance of capivasertib from plasma after oral administration; C_max_, maximum observed plasma (peak) drug concentration; C_trough_, trough plasma concentration; gCV%, geometric coefficient of variation; *N*, number of participants per visit; *n*, number of observations in analysis; PK, pharmacokinetic; t_½λz_, half-life associated with terminal slope (λz) of a semi-logarithmic concentration–time curve; t_max_, time to reach maximum observed plasma (peak) drug concentration
^a^*n*=16; ^b^*n*=15; ^c^*n*=14; ^d^*n*=13; ^e^*n*=20; ^f^*n*=12

**Supplementary Fig. 1** Patient disposition


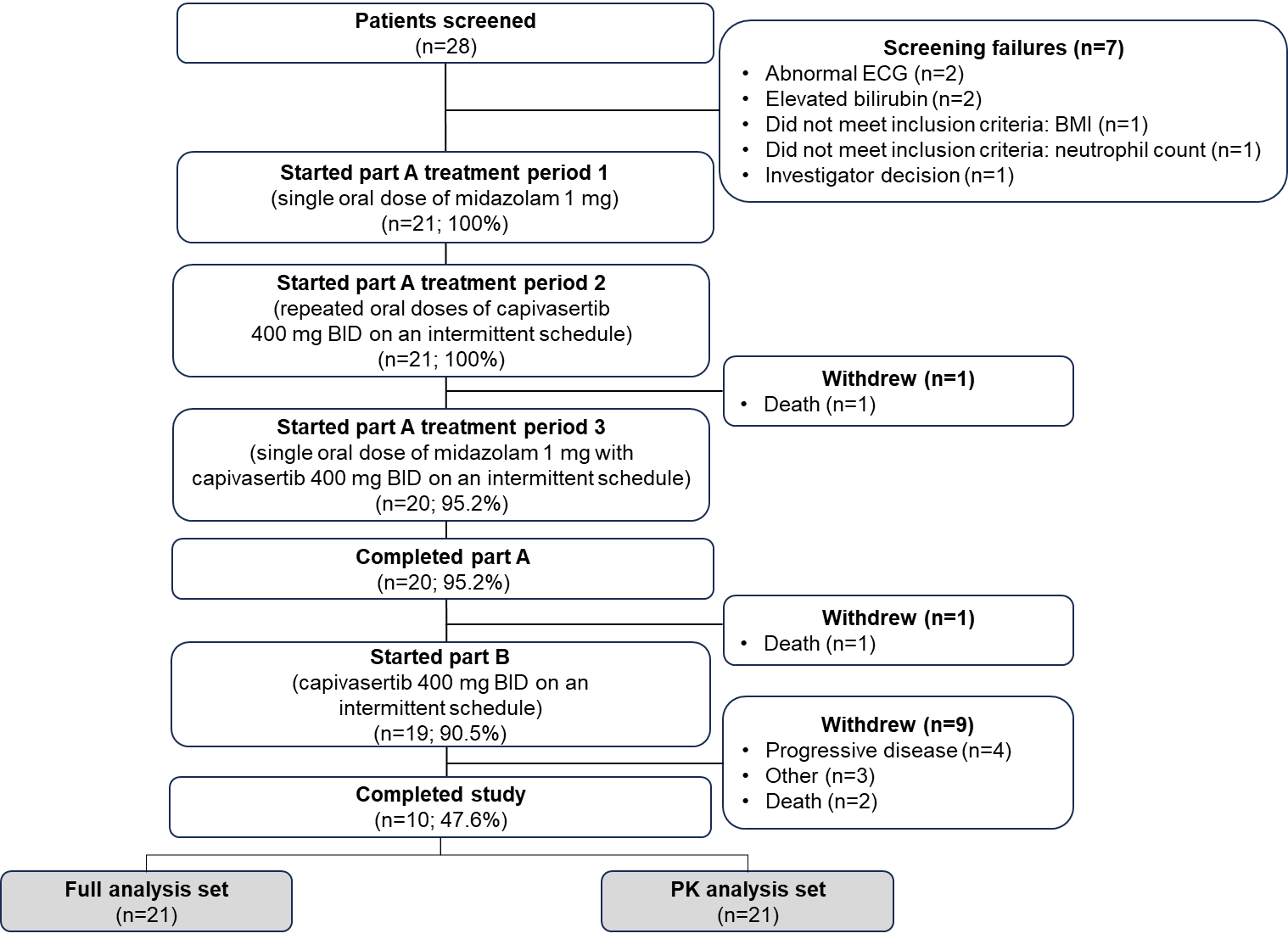


BID, twice daily; BMI, body mass index; ECG, electrocardiogram; PK, pharmacokinetic

**Supplementary Fig. 2** Plasma concentration of capivasertib and its metabolite, AZ14102143, over time on day 12 of cycle 1 in part A (PK analysis set)^a^


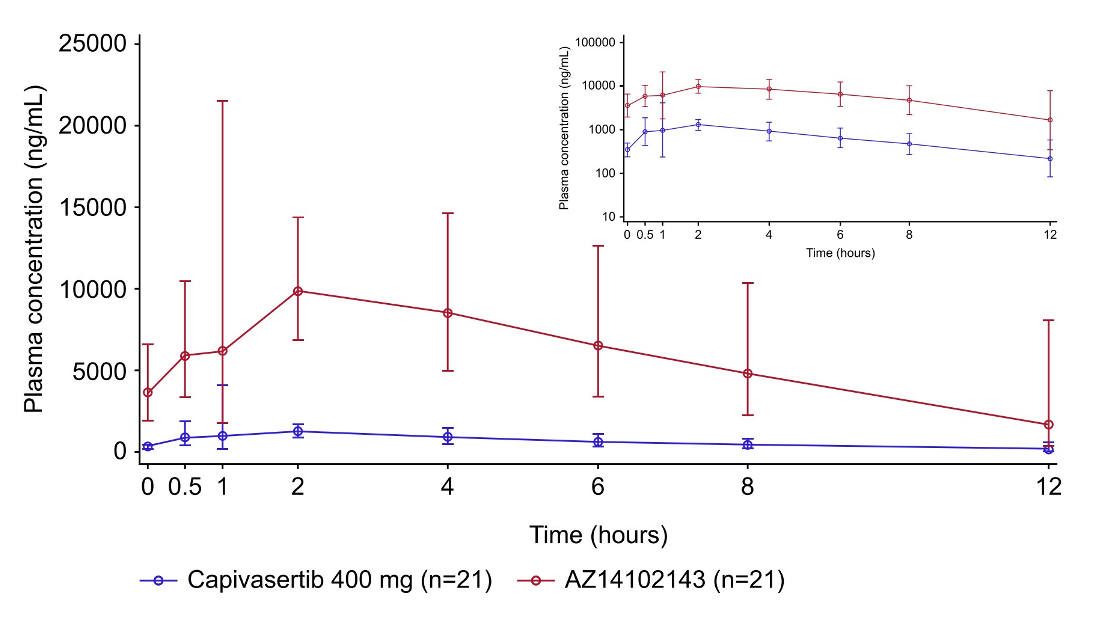
Points and error bars show the geometric mean ± geometric SD of plasma concentrations. Inset shows geometric mean values plotted on a semi-logarithmic scale. Treatment period 3 (day 12 of cycle 1): patients received a single oral dose of midazolam (1 mg) on day 12, corresponding to the 4^th^ day on capivasertib (400 mg BID on an intermittent schedule [4 days on/3 days off]). BID, twice daily; *n*, number of patients in the PK analysis set per treatment group; PK, pharmacokinetic; SD, standard deviation. ^a^A reliable elimination phase could not be determined for two capivasertib PK profiles and three metabolite PK profiles; these profiles were excluded
